# The -173 G/C Polymorphism of the MIF Gene and Inflammatory Bowel Disease Risk: A Meta-Analysis

**DOI:** 10.3390/ijms140611392

**Published:** 2013-05-28

**Authors:** Yongchun Shen, Shujin Guo, Ting Yang, Liuqun Jia, Lei Chen, Jing An, Tao Wang, Fuqiang Wen

**Affiliations:** Division of Internal Medicine, West China Hospital of Sichuan University, Chengdu 610041, China; E-Mails: shenyongchun1986@gmail.com (Y.S.); shujinguo@126.com (S.G.); yangting8506@163.com (T.Y.); huaxi198691@126.com (L.J.); resalex@126.com (L.C.); bunnysilent@163.com (J.A.); taowangwest@yahoo.com.cn (T.W.)

**Keywords:** inflammatory bowel disease, migration inhibitory factor, polymorphism, susceptibility, meta-analysis

## Abstract

The -173 G/C polymorphism in the macrophage migration inhibitory factor (MIF) gene has been implicated in susceptibility to inflammatory bowel disease (IBD), but the results are inconclusive. The present meta-analysis aimed to investigate the overall association between the -173 G/C polymorphism and IBD risk. We searched in Pubmed, and Embase for studies evaluating the association between the -173G/C gene polymorphism and IBD risk. Data were extracted and statistical analysis was performed using Revman 5.1 and STATA 12.0 software. A total of seven publications involving 4729 subjects (2282 IBD cases and 2447 controls) were included in this meta-analysis. Combined analysis revealed a clear association between this polymorphism and IBD susceptibility (OR = 1.48, 95% CI: 1.10–2.00, *p* = 0.009 for CC *vs*. CG + GG). Subgroup analysis by ethnicity showed that the IBD risk associated with the -173G/C gene polymorphism was significantly elevated among Asians (OR = 1.79, 95% CI: 1.08–2.96, *p* = 0.02), but not among Caucasians. Subgroup analysis by disease suggested that the -173G/C gene polymorphism is a risk factor for ulcerative colitis (OR = 1.62, 95% CI: 1.10–2.37, *p* = 0.01), but that it was not associated with Crohn’s disease. This meta-analysis suggests that the -173 G/C polymorphism in the macrophage MIF gene contributes to IBD susceptibility, specifically in Asian populations. Further studies are needed to validate these findings.

## 1. Introduction

Inflammatory bowel disease (IBD), including Crohn’s disease (CD) and ulcerative colitis (UC), refers to a heterogeneous group of chronic disorders that leads to cachexia. IBDs are essentially incurable, are of unknown etiology and typically follow a clinical course featuring remissions and relapses [1]. Incidence of IBD is increasing worldwide, and it places a heavy burden on patients because it reduces life quality and ability to work, in addition to increasing disability [2]. The etiology of IBD is complicated and the pathogenesis is poorly understood; nevertheless, growing evidence indicates that IBD results from an abnormal inflammatory response, in which genetic and environmental factors play important roles [3]. Numerous studies have investigated the association of genetic variants with IBD susceptibility [4], and among them, the macrophage migration inhibitory factor (MIF) gene has been highlighted.

MIF is an important pro-inflammatory cytokine that plays a critical role in regulating immune and inflammatory response [5]. Serum MIF levels were found to be significantly higher in patients with UC than in normal controls [6], and another study suggest that immunization with helper T epitope DNA-vaccine targeting MIF may be a useful approach for the treatment of colitis including inflammatory bowel diseases [7]. These findings suggest a potential role for MIF in IBD pathogenesis and treatment [8]; consistent with this notion, polymorphism in the MIF gene has been associated with susceptibility to inflammatory diseases, such as IBD [9]. Several studies have investigated whether the -173 G/C polymorphism in the macrophage MIF gene is associated with IBD risk, and the results have been inconsistent and inconclusive. Since pooled estimates based on meta-analysis have proven to be useful in determining the overall risk of certain IBD polymorphisms when results of individual studies are inconsistent [10], we decided to perform the present meta-analysis in order to clarify the association between the MIF -173G/C polymorphism and IBD risk.

## 2. Results and Discussion

### 2.1. Characteristics of Included Studies

A total of seven publications evaluating the association between the MIF -173G/C polymorphism and IBD risk were included in the meta-analysis, involving 4729 subjects (2282 IBDs cases and 2447 controls) [11–17]. In publications containing both a “CD group” and a “UC group”, each group was treated as a separate study in the meta-analysis. Among IBD cases, 1072 were diagnosed with CD and 1210 with UC. One study was excluded because the distribution of genotypes in the control group was inconsistent with HWE [18]. Four of the seven included publications described case-control studies involved Caucasians [12–14,17], while the remaining three publications involving Asians [11,15,16]. The characteristics of each case-control study are summarized in Table 1, and genotype and allele distributions for each case-control study are listed in Table 2.

### 2.2. Quantitative Data Synthesis

**Figure 1:**
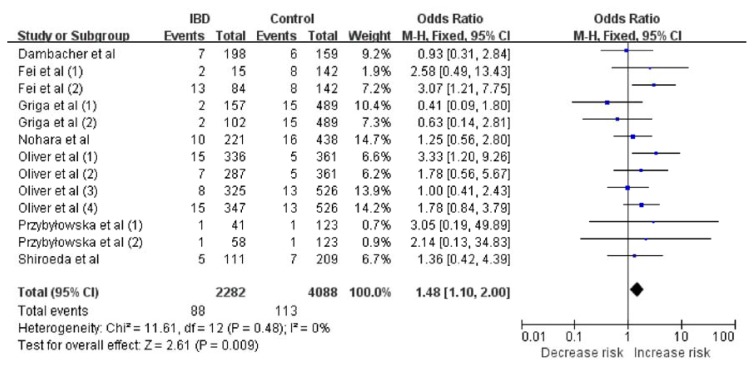
Meta-analysis using a fixed-effects model to evaluate the association between the MIF -173 G/C polymorphism and IBD risk (CC *vs*. CG + GG). The size of the square is proportional to the weight of each study; horizontal lines represent the 95% CI.

**Figure 2:**
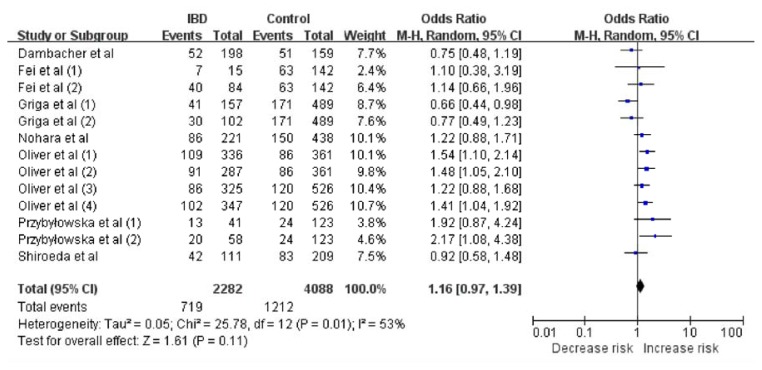
Meta-analysis using a random-effects model to evaluate the association between the MIF -173 G/C polymorphism and IBD risk (CC + CG *vs.* GG). The size of the square is proportional to the weight of each study; horizontal lines represent the 95% CI.

Given the proposal that the recessive model is the best genetic model for evaluating the association between the MIF-173G/C polymorphism in the MIF gene and IBD risk [19], we first analyzed the heterogeneity of CC *vs.* CG + GG in order to choose the most suitable calculation model. Across all included studies, χ^2^ was 11.61 and *p* = 0.48 for a random-effects model, and *I*^2^, another index of heterogeneity, was 0%. These findings suggested a lack of heterogeneity. Thus, we chose the fixed-effects model to synthesize the data. The pooled OR based on all studies was 1.48 (95% CI: 1.10–2.00), which was associated with a *Z* value of 2.61 (*p* = 0.009) (Figure 1). This suggested that CC homozygotic carriers have a higher risk of IBD than do CG and GG homozygotic individuals. We chose the random-effects model to synthesize the data according to the dominant genetic model. The pooled OR was 1.16 (95% CI: 0.97–1.39) and the associated *Z* value was 1.61 (*p* = 0.11) (Figure 2). These results suggested the possibility that CC homozygotic carriers and CG heterozygotic carriers have higher risk of IBD than do GG homozygotic individuals, but the results did not achieve statistical significance. Results for these and other genetic comparisons are summarized in Table 3.

### 2.3. Subgroup Analysis

Subgroup analysis by ethnicity, showed that, among the studies involving Asians [11,15,16], CC homozygotic carriers showed higher risk of IBD than did CG and GG homozygotic individuals, with an OR of 1.79 (95% CI: 1.08–2.96). Among the studies involving Caucasians [12–14,17], however, no association was found between the MIF -173G/C polymorphism and risk of IBD. When cases with CD or UC were analyzed in separate subgroups, no association was found between the -173G/C polymorphism in the MIF gene and risk of CD, while there was a significant association between the MIF -173G/C polymorphism and risk of UC in all the genetic models. Carriers of the C allele showed higher risk of UC than did carriers of the G allele, with an OR of 1.24 (95% CI: 1.09–1.41, *p* = 0.001).

### 2.4. Sensitivity Analysis and Publication Bias

**Figure 3:**
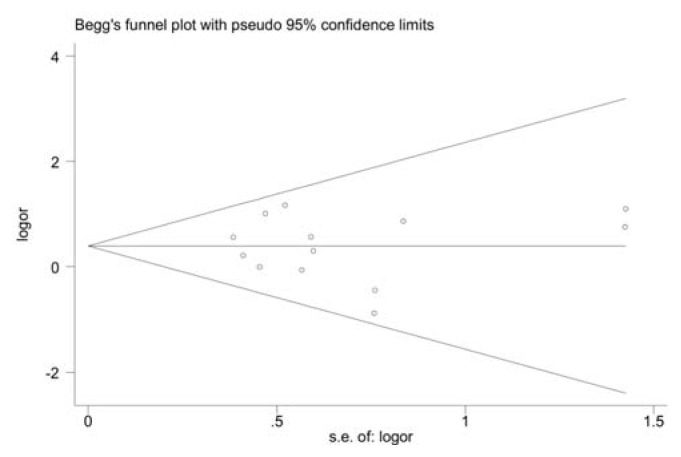
Begg’s funnel plot to detect publication bias in studies examining the MIF-173G/C polymorphism (CC *vs*. CG + GG).

To assess the stability of our findings, sensitivity analysis was performed by sequentially excluding each study. Statistically similar results were obtained after sequentially excluding each study, suggesting the stability of the results. Begg’s funnel plot and Egger’s test were used to assess publication bias. The shape of the funnel plots seemed symmetrical for the CC *vs*. CG + GG comparison genetic model, suggesting the absence of publication bias (Figure 3). Then, Egger’s test was performed to provide statistical evidence of funnel plots asymmetry. The results indicated a lack of publication bias of the present meta-analysis (*p* = 0.301).

### 2.5. Discussion

IBD refers to a group of chronic relapsing intestinal inflammatory diseases of unidentified causes. Taking epidemiological, genetic and immunological data together, indicates that IBD results from a complex interplay of genetic and environmental factors that produce an intestinal inflammatory response [20,21]. Gene variants may play an important role in the pathogenesis of IBD by altering protein function and individual’s susceptibility to disease [2,3,21]. Macrophage MIF has recently been reported to be a key response regulator that acts by directly activating immune cells or by participating in activation pathways initially triggered by other factors [4]. Increasing studies suggest that MIF plays an important role in the regulation of the innate immune response, and MIF may be involved in the pathogenesis of IBD and could be a potential target in the treatment of IBD [8], but studies of the association between the MIF -173G/C polymorphism and risk of IBD have given inconsistent and inconclusive results. Therefore, we performed this meta-analysis to clarify the relationship between this polymorphism and susceptibility to IBD. To our knowledge, it is the first meta-analysis of the possible association between the MIF-173G/C polymorphism and IBD risk.

A total of seven publications describing thirteen case-control studies in Asians and Caucasians were included in this meta-analysis. The effects of dominant/recessive models and of allele frequency were all estimated. In addition, the consistency of genetic effects across different ethnicities and IBD types was investigated. Our findings support the notion that, among Asians, the MIF -173G/C polymorphism plays a role in the development of IBD, and that the homozygotic CC genotype may be a risk factor for IBD.

Our data indicate a strong ethnic bias in the association between the MIF -173G/C polymorphism and risk of IBD. We found the polymorphism to be a risk factor for IBD among Asians, with CC homozygotic carriers at higher risk of IBD than CG and GG homozygotic indivividuals. Among Caucasians, however, we found no evidence of an association between the MIF -173G/C polymorphism and risk of IBD. We also identified a disease-specific bias in the association. While we did not find any evidence of an association between the MIF -173G/C polymorphism and risk of CD, we did find that the polymorphism may be a risk factor for UC, suggesting that the associations between MIF -173G/C polymorphism and IBD risk are ethnicity and disease specific. These findings are consistent with a recent study suggesting that, although the etiology of CD and UC may overlap, the two diseases appear to have different genetic risk profiles [22].

Our finding that the -173G/C polymorphism is a genetic risk factor for IBD in Asians but apparently not Caucasians, suggests population-specific genetic differences in IBD pathogenesis. Therefore, depending on the population, IBD may be associated with different genes, different loci within the same gene, and/or different polymorphisms at the same locus. Indeed, a systematic review of genes associated with susceptibility to IBD in Asian populations found that genetic risk factors differed between Asians and Caucasians [23]. Those authors proposed that new mutations and susceptibility genes identified in Asian IBD patients provide an opportunity to explore new disease-associated mechanisms in this population of rising incidence [23]. Since that review did not examine the macrophage MIF gene, the present meta-analysis extends our appreciation of the complex etiology and genetic risk factors of IBD.

Further research is needed to examine not only the genetic risk factors of IBD, but the environmental risk factors as well. Based on available evidence, we cannot exclude the possibility that environmental risk factors explain at least part of the ethnic bias observed here in the association between the MIF -173G/C polymorphism and IBD risk. Therefore further work is essential to tease apart the relative contributions of genes and environment. In addition, to take consideration of population differences will be particularly informative, susceptibility genes identified in IBD patients with different ethnicities provide an opportunity to explore new mechanisms of disease that are specific in different population. What’s more, strongly suggesting that blockade of MIF bioactivities by either neutralizing anti-MIF antibodies or antagonists prevents an inflammatory cytokine cascade, which strongly suggests that targeting MIF may be effective for treating IBD [24]. Thus, a comprehensive understanding of genetic, epigenetic, environmental, and clinical factors may not only improve our understanding of the mechanisms of IBD, but also lead to more effective prevention and treatment [25].

The findings in this meta-analysis should be interpreted with caution because of several limitations. First, a relatively small number of studies and subjects were included in this meta-analysis, which may reduce the statistical power for identifying possible associations between the MIF -173G/C polymorphism and IBD risk. In particular, the apparent lack of association between this polymorphism and disease risk in Caucasians and in patients with CD should be verified in larger-scale studies. Second, the included publications were limited to Asian and Caucasian populations, so future work should examine other populations, such as Latinos, especially given substantial evidence of ethnic bias in the -173G/C polymorphism. Third, although we did not set any language restrictions during our literature searching, we included only English-language publications in the meta-analysis. It is possible that our results would be different if they included the findings of unpublished studies or of relevant studies published in other languages.

## 3. Experimental Section

### 3.1. Literature Search

Two authors independently performed systematic searches of Pubmed and Embase databases in January 2013 to identify studies examining the association between the -173G/C polymorphism in the MIF gene and IBD risk. Search terms were as follows: “IBD or inflammatory bowel disease or Crohn’s disease or ulcerative colitis” in combination with “migration inhibitory factor” in combination with “polymorphism or variant or mutation”. The reference lists of identified studies and review articles were manually searched to find additional relevant publications.

### 3.2. Study Selection

Studies were included in the meta-analysis if they satisfied the following inclusion criteria: (1) they evaluated the potential association between the MIF gene -173G/C polymorphism and IBD risk; (2) they were case-control studies; (3) genotype distributions were available for cases and controls in order to estimate an odds ratio (OR) with 95% confidence interval (CI); (4) the distribution of genotypes in the control group was consistent with Hardy-Weinberg equilibrium (HWE). Abstracts, reviews, and studies in which genotype frequencies were not reported were excluded; and (5) when publications involved the same or overlapping data sets, only the study with the largest number of participants was included.

### 3.3. Data Extraction

Two reviewers independently extracted data from the final set of included studies. The following data were extracted: the name of the first author, year of publication, country of origin, ethnicity, sample size, IBD diagnosis, genotyping method, and genotype frequencies in IBD cases and controls.

### 3.4. Statistical Analysis

The strength of the association between the MIF -173G/C polymorphism and risk of IBD was assessed using ORs and 95% CIs. The significance of the pooled OR was determined using the *Z*-test and *p* < 0.05 was considered statistically significant. First, we evaluated the dominant model (CC + CG *vs*. GG) and recessive model (CC *vs*. CG + GG), followed by the additive model (CC *vs.* GG). We also estimated the association based on allelic contrast (C *vs*. G). To evaluate whether the association showed any ethnicity- or disease-specific effects, we analyzed the data for separate subgroups defined by ethnicity and diagnosis with CD or UC.

Heterogeneity was evaluated using a χ^2^-based Q statistic and *I*^2^ statistic, with *p* < 0.10 considered statistically significant. When *p* ≥ 0.10, the pooled OR of each study was calculated using a fixed-effects model; otherwise, a random-effects model was used.

Publication bias was assessed using Begg’s funnel plots and Egger’s test [26,27]. Sensitivity analysis was performed by sequentially excluding individual studies and recalculating the results. Pearson’s ×2 test was used to determine whether the observed frequencies of genotypes in control group conformed to the HWE[28]. All statistical tests were performed using Revman 5.1 and STATA 12.0 software.

## 4. Conclusions

To the best of our knowledge, this is the first meta-analysis to assess the relationship between the MIF-173G/C polymorphism and IBD risk. Our results suggest that the -173G/C polymorphism is a risk factor for IBD in Asians but not in Caucasians. Large well-designed, multi-center epidemiological studies should be carried out in these and other ethnic populations to confirm our findings.

## Figures and Tables

**Table 1:** Characteristics of included studies.

Author	Year	Country	Ethnicity	Diagnosis	Cases	Controls	Genotyping method	HWE
Dambacher *et al.* [14]	2007	Germany	Caucasian	Crohn’s disease	198	159	PCR	Y
Fei *et al.* [15]	2008	China	Asian	Crohn’s disease	15	142	RFLP-PCR	Y
Fei *et al.* [15]	2008	China	Asian	Ulcerative colitis	84	142	RFLP-PCR	Y
Griga *et al.* [12]	2007	Germany	Caucasian	Crohn’s disease	157	489	RFLP-PCR	Y
Griga *et al.* [12]	2007	Germany	Caucasian	Ulcerative colitis	102	489	RFLP-PCR	Y
Nohara *et al.* [11]	2004	Japan	Asian	Ulcerative colitis	221	438	Tetraprimer-PCR	Y
Oliver *et al.* [13]	2007	Spain	Caucasian	Crohn’s disease	336	361	PCR	Y
Oliver *et al.* [13]	2007	Spain	Caucasian	Ulcerative colitis	287	361	PCR	Y
Oliver *et al.* [13]	2007	Spain	Caucasian	Crohn’s disease	325	526	PCR	Y
Oliver *et al.* [13]	2007	Spain	Caucasian	Ulcerative colitis	347	526	PCR	Y
Przybyłowska *et al.* [17]	2011	Poland	Caucasian	Crohn’s disease	41	123	RFLP-PCR	Y
Przybyłowska *et al.* [17]	2011	Poland	Caucasian	Ulcerative colitis	58	123	RFLP-PCR	Y
Shiroeda *et al.* [16]	2010	Japan	Asian	Ulcerative colitis	111	209	SSCP-PCR	Y

Note: PCR: Polymerase chain reaction; RFLP: Restriction fragment length polymorphism; SSCP: Single strand conformation polymorphism; HWE: Hardy-Weinberg equilibrium; Y: Yes.

**Table 2:** Distribution of migration inhibitory factor (MIF) genotype and allele among inflammatory bowel disease (IBD) patients and controls.

Author	IBD			Control			IBD		Control	
			
	GG	GC	CC	GG	GC	CC	G	C	G	C
Dambacher *et al.* [14]	146	45	7	108	45	6	335	59	261	57
Fei *et al.* [15]	8	5	2	79	55	8	21	9	213	71
Fei *et al.* [15]	44	27	13	79	55	8	115	53	213	71
Griga *et al.* [12]	116	39	2	318	156	15	271	43	792	186
Griga *et al.* [12]	72	28	2	318	156	15	172	32	792	186
Nohara *et al.* [11]	135	76	10	288	134	16	346	96	710	166
Oliver *et al.* [13]	227	94	15	275	81	5	548	124	631	91
Oliver *et al.* [13]	196	84	7	275	81	5	476	98	631	91
Oliver *et al.* [13]	239	78	8	406	107	13	556	94	919	133
Oliver *et al.* [13]	245	87	15	406	107	13	577	117	919	133
Przybyłowska *et al.* [17]	28	12	1	99	23	1	68	14	221	25
Przybyłowska *et al.* [17]	38	19	1	99	23	1	95	21	221	25
Shiroeda *et al.* [16]	69	37	5	126	76	7	175	47	328	90

**Table 3:** Summary of different comparative results.

	CC + CG *vs*. GG		CC *vs*. CG + GG		CC *vs*.GG		C *vs*.G	
				
	OR (95% CI)	p*	OR (95% CI)	p*	OR (95% CI)	p*	OR (95% CI)	p*
Total	1.16 (0.97–1.39)	0.11	**1.48 (1.10**–**2.00)**	0.009	**1.50 (1.12**–**2.03)**	0.007	1.18 (1.00–1.38)	0.05
Subgroup by Ethnicity								
Caucasian	1.18 (0.93–1.51)	0.17	1.35 (0.94–1.95)	0.11	1.38 (0.96**–**2.00)	0.08	1.18 (0.94–1.47)	0.16
Asian	1.12 (0.88–1.42)	0.36	**1.79 (1.08–2.96)**	0.02	**1.78 (1.06–2.98)**	0.03	1.18 (0.97–1.43)	0.11
Subgroup by diagnosis								
Crohn’s disease	1.08 (0.77–1.51)	0.64	1.31 (0.81–2.09)	0.27	1.31 (0.82–2.09)	0.27	1.11 (0.81–1.52)	0.52
Ulcerative colitis	**1.24(1.06–1.44)**	0.006	**1.62 (1.10–2.37)**	0.01	**1.65 (1.13–2.43)**	0.01	**1.24 (1.09–1.41)**	0.001

Note: The bold values mean that their association is significant,

* *p* value for *Z* test.
